# Myocardial structural and functional changes in cardiac amyloidosis: insights from a prospective observational patient registry

**DOI:** 10.1093/ehjci/jead188

**Published:** 2023-08-07

**Authors:** Franz Duca, René Rettl, Christina Kronberger, Christina Binder, Christopher Mann, Fabian Dusik, Lore Schrutka, Daniel Dalos, Begüm Öztürk, Theresa Marie Dachs, Bernhard Cherouny, Luciana Camuz Ligios, Hermine Agis, Renate Kain, Matthias Koschutnik, Carolina Donà, Roza Badr-Eslam, Johannes Kastner, Dietrich Beitzke, Christian Loewe, Christian Nitsche, Christian Hengstenberg, Andreas Anselm Kammerlander, Diana Bonderman

**Affiliations:** Department of Internal Medicine II, Division of Cardiology, Medical University of Vienna, Waehringer Guertel 18-20, 1090 Vienna, Austria; Department of Internal Medicine II, Division of Cardiology, Medical University of Vienna, Waehringer Guertel 18-20, 1090 Vienna, Austria; Department of Internal Medicine II, Division of Cardiology, Medical University of Vienna, Waehringer Guertel 18-20, 1090 Vienna, Austria; Department of Internal Medicine II, Division of Cardiology, Medical University of Vienna, Waehringer Guertel 18-20, 1090 Vienna, Austria; Department of Internal Medicine II, Division of Cardiology, Medical University of Vienna, Waehringer Guertel 18-20, 1090 Vienna, Austria; Department of Internal Medicine II, Division of Cardiology, Medical University of Vienna, Waehringer Guertel 18-20, 1090 Vienna, Austria; Department of Internal Medicine II, Division of Cardiology, Medical University of Vienna, Waehringer Guertel 18-20, 1090 Vienna, Austria; Department of Internal Medicine II, Division of Cardiology, Medical University of Vienna, Waehringer Guertel 18-20, 1090 Vienna, Austria; Division of Cardiology, Favoriten Clinic, Kundratstraße 3, 1100 Vienna, Austria; Department of Internal Medicine II, Division of Cardiology, Medical University of Vienna, Waehringer Guertel 18-20, 1090 Vienna, Austria; Department of Internal Medicine II, Division of Cardiology, Medical University of Vienna, Waehringer Guertel 18-20, 1090 Vienna, Austria; Department of Internal Medicine II, Division of Cardiology, Medical University of Vienna, Waehringer Guertel 18-20, 1090 Vienna, Austria; Department of Internal Medicine I, Division of Hematology, Medical University of Vienna, Vienna, Austria; Clinical Institute of Pathology, Medical University of Vienna, Vienna, Austria; Department of Internal Medicine II, Division of Cardiology, Medical University of Vienna, Waehringer Guertel 18-20, 1090 Vienna, Austria; Department of Internal Medicine II, Division of Cardiology, Medical University of Vienna, Waehringer Guertel 18-20, 1090 Vienna, Austria; Department of Internal Medicine II, Division of Cardiology, Medical University of Vienna, Waehringer Guertel 18-20, 1090 Vienna, Austria; Department of Internal Medicine II, Division of Cardiology, Medical University of Vienna, Waehringer Guertel 18-20, 1090 Vienna, Austria; Department of Bioimaging and Image-Guided Therapy, Division of Cardiovascular and Interventional Radiology, Medical University of Vienna, Vienna, Austria; Department of Bioimaging and Image-Guided Therapy, Division of Cardiovascular and Interventional Radiology, Medical University of Vienna, Vienna, Austria; Department of Internal Medicine II, Division of Cardiology, Medical University of Vienna, Waehringer Guertel 18-20, 1090 Vienna, Austria; Department of Internal Medicine II, Division of Cardiology, Medical University of Vienna, Waehringer Guertel 18-20, 1090 Vienna, Austria; Department of Internal Medicine II, Division of Cardiology, Medical University of Vienna, Waehringer Guertel 18-20, 1090 Vienna, Austria; Department of Internal Medicine II, Division of Cardiology, Medical University of Vienna, Waehringer Guertel 18-20, 1090 Vienna, Austria; Division of Cardiology, Favoriten Clinic, Kundratstraße 3, 1100 Vienna, Austria

**Keywords:** cardiac amyloidosis, transthyretin amyloidosis, light chain amyloidosis, T1 mapping, extracellular volume, cardiac magnetic resonance imaging

## Abstract

**Aims:**

The pathophysiological hallmark of cardiac amyloidosis (CA) is the deposition of amyloid within the myocardium. Consequently, extracellular volume (ECV) of affected patients increases. However, studies on ECV progression over time are lacking. We aimed to investigate the progression of ECV and its prognostic impact in CA patients.

**Methods and results:**

Serial cardiac magnetic resonance (CMR) examinations, including ECV quantification, were performed in consecutive CA patients. Between 2012 and 2021, 103 CA patients underwent baseline and follow-up CMR, including ECV quantification. Median ECVs at baseline of the total (*n* = 103), transthyretin [(ATTR) *n* = 80], and [light chain (AL) *n* = 23] CA cohorts were 48.0%, 49.0%, and 42.6%, respectively. During a median period of 12 months, ECV increased significantly in all cohorts [change (Δ) +3.5% interquartile range (IQR): −1.9 to +6.9, *P* < 0.001; Δ +3.5%, IQR: −2.0 to +6.7, *P* < 0.001; and Δ +3.5%, IQR: −1.6 to +9.1, *P* = 0.026]. Separate analyses for treatment-naïve (*n* = 21) and treated (*n* = 59) ATTR patients revealed that the median change of ECV from baseline to follow-up was significantly higher among untreated patients (+5.7% vs. +2.3%, *P* = 0.004). Survival analyses demonstrated that median change of ECV was a predictor of outcome [total: hazard ratio (HR): 1.095, 95% confidence interval (CI): 1.047–1.0145, *P* < 0.001; ATTR: HR: 1.073, 95% CI: 1.015–1.134, *P* = 0.013; and AL: HR: 1.131, 95% CI: 1.041–1.228, *P* = 0.003].

**Conclusion:**

The present study supports the use of serial ECV quantification in CA patients, as change of ECV was a predictor of outcome and could provide information in the evaluation of amyloid-specific treatments.

## Graphical Abstract

**Graphical Abstract:**
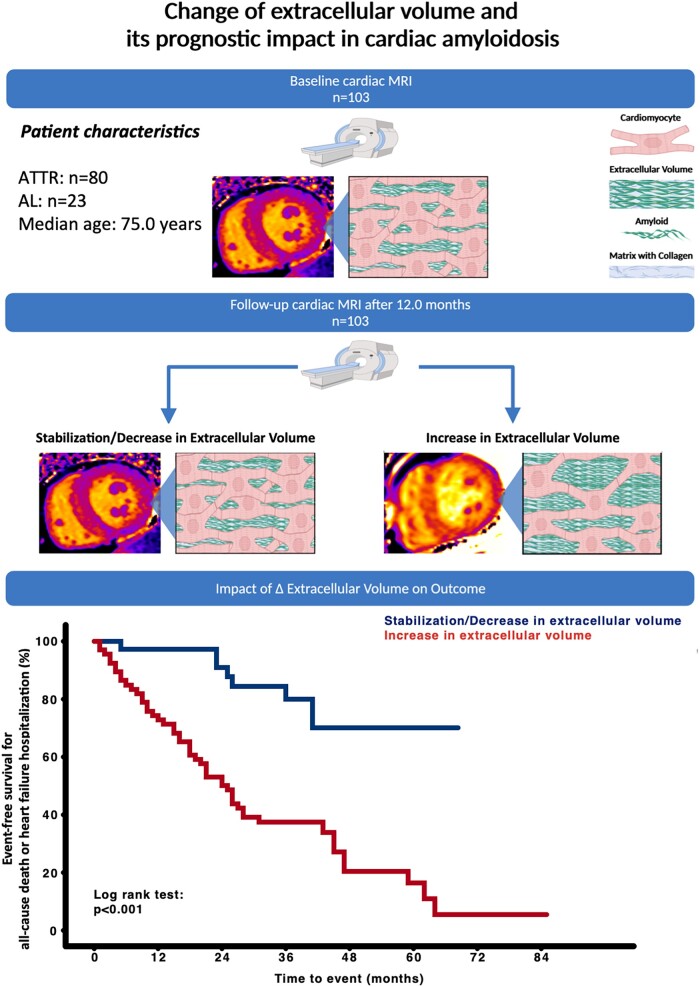
Change of extracellular volume and its prognostic impact in cardiac amyloidosis. Change in (Δ) extracellular volume measured by cardiac magnetic resonance T1 mapping correlates with outcome in patients with cardiac amyloidosis.

## Introduction

Within the last decade, cardiac amyloidosis (CA) has been acknowledged as a significant cause of heart failure (HF) and patients who are affected face a dismal prognosis, especially in later stages of the disease.^1^ The pathophysiological hallmark of CA is the deposition of misfolded proteins (amyloid) within the myocardial extracellular space, which in turn leads to an expansion of the extracellular volume (ECV).^2,3^ The gold standard for ECV quantification is histological assessment of amyloid burden in endomyocardial biopsies (EMB). However, due to associated procedural risks and the patchy distribution of amyloid within the myocardium, EMB is not feasible for serial assessment of ECV. In recent years, cardiac magnetic resonance (CMR) T1 mapping has been validated as an accurate, non-invasive method for ECV quantification in various cardiac diseases including CA.^4,5^

Despite the fact that ECV expansion is the central pathophysiologic mechanism in CA, our knowledge on ECV changes over the course of the disease and its prognostic implications is limited.^6,7^

We therefore performed sequential ECV quantification using CMR T1 mapping and evaluated the prognostic impact of its changes on outcome in treated, as well as treatment-naïve, transthyretin (ATTR) and light chain (AL) amyloidosis patients.

## Methods

### Setting and study design

The present study was conducted in accordance with good clinical practice as outlined in the declaration of Helsinki and performed at the Department of Cardiology at the Medical University of Vienna, which includes a dedicated amyloidosis outpatient clinic, as well as a multimodality imaging laboratory. The ethics committee of the Medical University of Vienna (EK# 796/2010) approved the study, and written informed consent was obtained from all patients before enrolment.

### Diagnosis of cardiac transthyretin amyloidosis and light chain amyloidosis

Diagnosis of ATTR and AL CA was made in accordance with current recommendations which are provided as Supplementary data online.^8^

### Study schedule

Baseline and follow-up visits of study participants consisted of CMR imaging with T1 mapping. Clinical and laboratory assessment included New York Heart Association (NYHA) class, 6-min walk distance (6-MWD), *N*-terminal pro brain natriuretic peptide (NT-proBNP), troponin T, and estimated glomerular filtration rate.

### Cardiac magnetic resonance imaging

CMR examinations were performed on a 1.5 Tesla scanner (MAGNETOM Avanto, Siemens Healthcare GmbH, Erlangen, Germany) according to standard protocols including late gadolinium enhancement imaging (0.1 mmoL/kg gadobutrol; Gadovist, Bayer Vital GmbH, Leverkusen, Germany) and T1 mapping using the modified Look-Locker inversion (MOLLI) sequence. Supplementary data online, *Figure S1*, depicts myocardial and blood pool regions of interest on T1 maps. The detailed CMR acquisition protocols and parameters are provided as Supplementary data online.

### Outcome measures

The primary outcome measure was a combination of all-cause death or hospitalization for HF.

HF-related hospitalizations were defined as events accompanied by worsening of dyspnoea, and/or weight gain, and/or peripheral oedema requiring admission to hospital and/or intravenous diuretic therapy. Outcome events were documented during follow-up at our outpatient clinic or telephone visits and were also retrieved from electronic medical records, as well as the Austrian death registry.

### Haematological response in light chain amyloidosis

Haematological response to treatment in AL CA patients was defined according to current recommendations with detailed definitions provided as Supplementary data online.^9^

### Statistical analysis

Continuous variables are expressed as median and interquartile range (IQR). Categorical variables are presented as numbers and percent. Continuous variables were compared using the Wilcoxon signed-rank for paired and the Mann–Whitney *U* test for non-paired variables. McNemar’s test was used for paired categorical parameters. Kaplan–Meier plots and respective log rank tests were used to verify time-dependent discriminative power of parameters of interest. Cox regression models were calculated in order to assess the effect of CMR parameters on event-free survival. For outcome analyses of T1 mapping parameters, the median individual change was used. IBM SPSS version 27.0 and STAT 16 were used for statistical analysis. The graphical abstract was created with Biorender.com. A *P* value of ≤0.05 was set as the level of significance. A more detailed explanation of the statistical methods is provided in the Supplementary data online.

## Results

**Figure 1:**
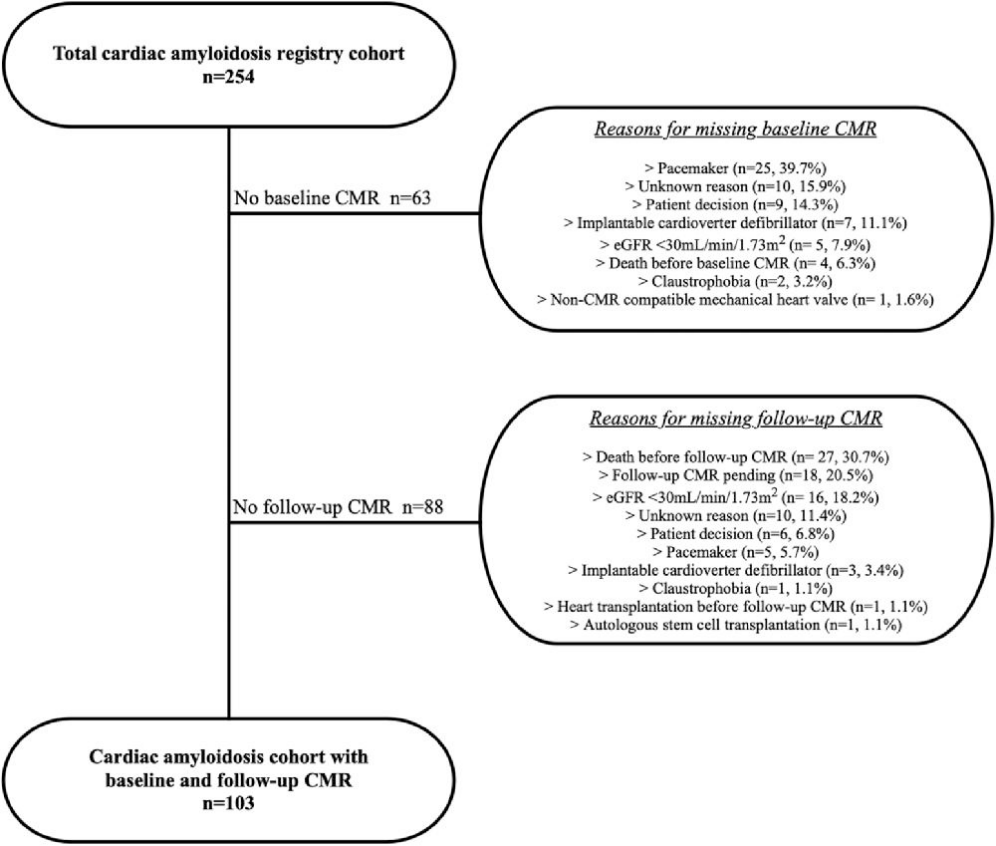
Patient flow chart. Reasons for missing baseline and follow-up cardiac magnetic resonance imaging studies among the total cardiac amyloidosis registry cohort. CMR, cardiac magnetic resonance imaging; eGFR, estimated glomerular filtration rate).

Between March 2012 and January 2021, 254 patients with CA were enrolled into our prospective registry. A total of 151 patients were excluded from the present analysis because either baseline (*n* = 63) or follow-up CMR (*n* = 88) was not performed. Reasons for exclusion are depicted in *Figure 1*. Notably, no patient had to be excluded due to insufficient CMR image quality.

The final cohort (*n* = 103) for the present study consisted of 70 (68.0%) patients with wild-type ATTR, 10 (9.7%) with variant ATTR (Val40Ile, *n* = 1; Thr80Arg, *n* = 1; Thr69ile, *n* = 1; Val50Met, *n* = 3; and His80Arg, *n* = 4) and 23 (22.3%) with AL CA who underwent baseline as well as follow-up CMR imaging.

Median time difference between baseline and follow-up CMR was 12.0 month (IQR: 9.0–21.0).

### Baseline and follow-up parameters of the total, transthyretin, and light chain cardiac amyloidosis cohorts

The total CA study cohort (*Table 1* and Supplementary data online, *Table S1*) was elderly with a median age of 75.0 years and predominantly male (79.6%). During follow-up, NT-proBNP (2222 pg/mL) and troponin T (0.051 ng/mL) levels increased, whereas 6-MWD (323 m) and eGFR (54.0 mL/min/1.73 m^2^) decreased.

**Table 1:** Baseline and follow-up characteristics of the complete cardiac amyloidosis cohort

Variable	Baseline	Follow-up	*P* value
	(*n* = 103)	(*n* = 103)	
**Clinical parameters**			
Age, years	75.0 (68.0–79.0)	77.0 (70.0–81.0)	<0.001
Sex, male gender, *n*	82 (79.6)	82 (79.6)	1.000
NYHA functional class ≥ III, *n*	36 (35.0)	25 (24.3)	0.054
6-min walk test, m	400 (296–480)	402 (323–486)	0.858
*N*-terminal pro brain natriuretic peptide, pg/mL	1917 (787–3542)	2222 (810–3502)	0.400
Troponin T, ng/mL	0.043 (0.029–0.065)	0.051 (0.028–0.071)	0.093
eGFR, mL/min/1.73m^2^	56.5 (42.4–75.0)	54.0 (43.0–68.7)	0.019
**Cardiac magnetic resonance imaging parameters**			
Myocardial native T1 time, ms	1099 (1057–1139)	1107 (1077–1144)	0.006
Individual change of myocardial T1 time, %		+12.0 (−17.5 to +36.3)	n.a
Extracellular volume, %	48.0 (40.0–55.3)	50.5 (43.1–59.5)	<0.001
Individual change of extracellular volume, %		+3.5 (−1.9 to +6.9)	n.a
Interventricular septum, mm	18.0 (15.0–21.0)	18.4 (16.0–22.0)	0.695
Left ventricular mass, g	194 (154–229)	195 (150–231)	0.449
Left atrial area, cm^2^	31.0 (26.0–37.0)	32.0 (28.0–36.0)	0.591
Right atrial area, cm^2^	30.0 (25.0–36.0)	30.0 (26.8–35.0)	0.241
Left ventricular global longitudinal strain, %	−12.6 (−15.3 to −10.1)	−12.3 (−15.4 to −9.5)	0.348
Left ventricular ejection fraction, %	56.0 (47.0–62.0)	50.6 (45.0–60.0)	0.009
Left ventricular cardiac output, L/min	5.7 (4.9–6.5)	5.8 (4.9–6.4)	0.829
Left ventricular end-diastolic volume, mL	159 (139–189)	166 (150–205)	<0.001
Right ventricular ejection fraction, %	49.0 (41.0–58.0)	45.9 (39.0–53.0)	<0.001
Right ventricular cardiac output, L/min	5.3 (4.5–6.4)	5.3 (4.4–6.3)	0.745
Right ventricular end-diastolic volume, mL	171 (142–191)	177 (154–209)	<0.001
Pulmonary artery, mm	28.0 (25.0–31.0)	28.0 (25.0–31.0)	0.786
Pleural effusion, *n*	28 (27.2)	35 (34.0)	0.189
Pericardial effusion, *n*	43 (41.7)	44 (42.7)	1.000

NYHA, New York Heart Association; eGFR, estimated glomerular filtration rate; n.a, not applicable. Numbers in brackets are % for dichotomous and IQR for continuous variables.

Magnetic resonance imaging. T0 was date of follow-up CMR.

**Figure 2:**
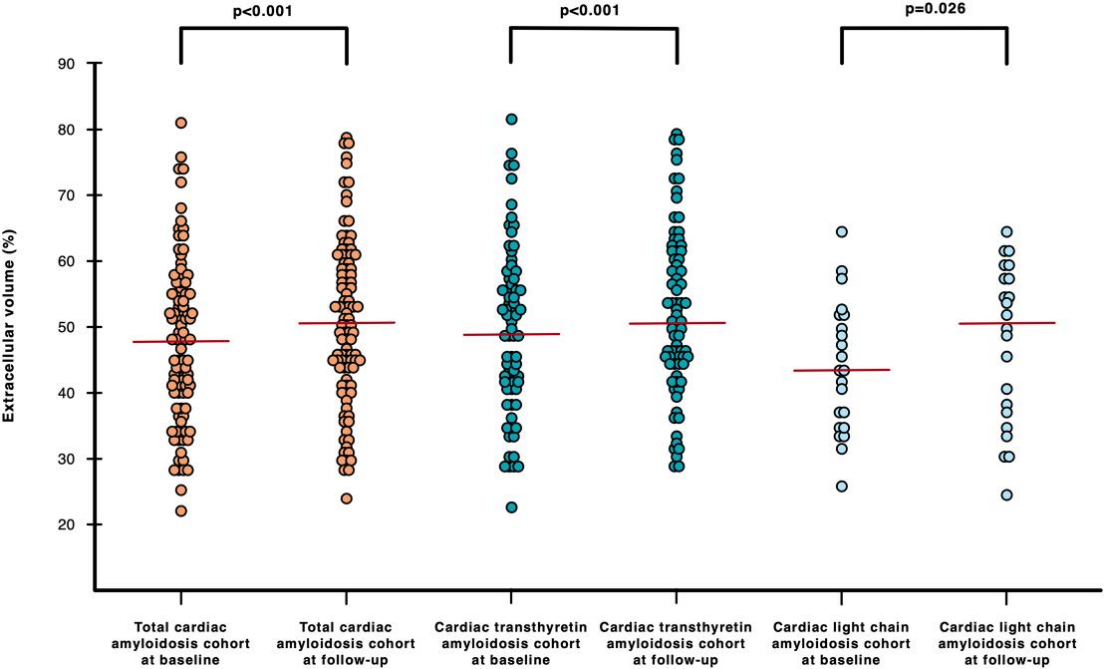
Dot plots showing extracellular volume (ECV) at baseline and follow-up for the total (orange), transthyretin (turquoise), and light chain (light blue) cardiac amyloidosis cohorts. ECV at baseline and follow-up for the total cardiac amyloidosis cohort were 48.0% (IQR: 40.0–55.3) and 50.5% (IQR: 43.1–59.5 *P* < 0.001). ECV at baseline and follow-up for the cardiac transthyretin amyloidosis were 48.0% (IQR: 40.0–55.3) and follow-up 50.5% (IQR: 43.1–59.5, *P* < 0.001). ECV at baseline and follow-up for the cardiac light chain amyloidosis cohort were 42.6% (IQR: 34.4–51.0) and 50.6% (IQR: 35.7–57.4, *P* = 0.028). Red lines indicate median.

**Figure 3:**
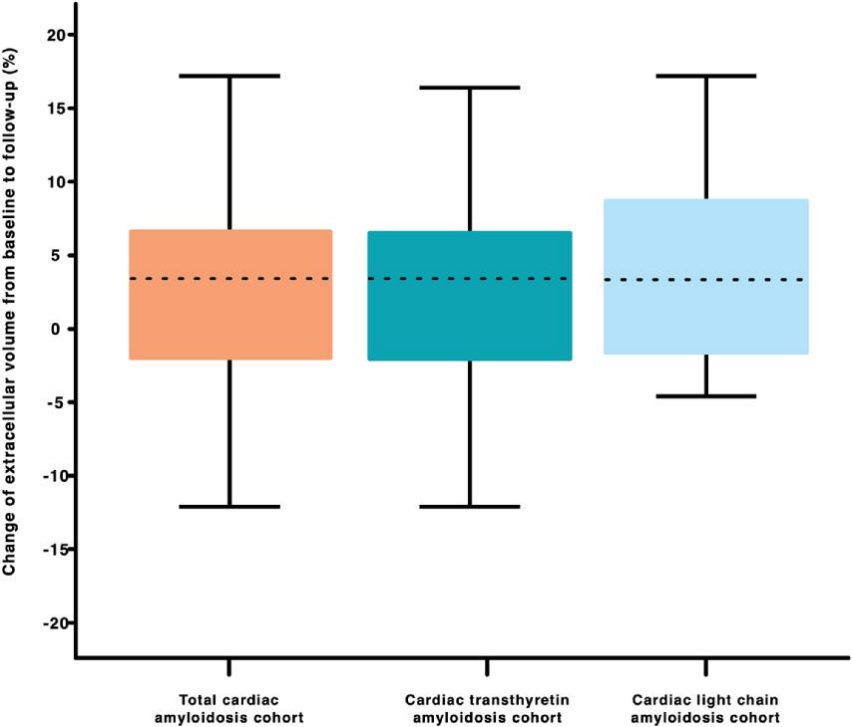
Median change of extracellular volume in the total (orange), transthyretin (turquoise), and light chain cardiac amyloidosis (light blue) cohorts. Median changes of extracellular volume from baseline to follow-up in the total, transthyretin, and light chain cardiac amyloidosis cohort were +3.5% (IQR: −1.9 to +6.9), +3.5% (IQR: −2.0 to +6.7), and +3.5% (IQR: −1.6 to +9.1).

CA patients showed characteristic myocardial changes on CMR imaging. ECV (48.0%) and native T1 times (1099 ms) were elevated. LVs were significantly hypertrophied [intraventricular septum (IVS) thickness: 18.0 mm, LV mass of 194 g, while LV ejection fraction (EF) was preserved (56.0%)]. During the follow-up period, ECV (50.5%, *P* < 0.001; *Figure 2* and Supplementary data online, *Figure S2*) and native T1 time (1107 ms, *P* = 0.006) increased significantly. Change of (Δ) ECV and T1 time were +3.5% (*Figure 3*) and 12.0 ms, respectively. A decreasing ECV was found in 35 (34.0%) patients. No correlation was found between Δ of ECV and time interval between CMRs (*r* = 0.079, *P* = 430). Furthermore, we were able to detect a decrease in LVEF (*P* = 0.009) as well as RVEF [45.9% (*P* < 0.001)].

The ATTR CA cohort [see Supplementary data online, *Table S2*; (*n* = 80)] was mostly male (82.5%) with a median age of 76.0 years. According to the ATTR staging system proposed by Gillmore and colleagues,^1^ 61.3% patients were in stage 1, 22.5% were in stage 2, and 15.0% in stage 3.

ECV (49.0%) and T1 times (1100 ms) were clearly elevated and accompanied by LV thickening (IVS, 19.0 mm; LV mass, 197 g). Over the course of our study, ECV (*P* < 0.001; *Figure 2* and Supplementary data online, *Figure S2*), native T1 time (*P* = 0.034), LV end-diastolic volume [(EDV), *P* < 0.001], and RVEDV (*P* < 0.001) increased, whereas LVEF (*P* = 0.033) and RVEF (*P* = 0.009) decreased. Median Δ of ECV in the ATTR cohort was +3.5% (*Figure 3*), while ECV decreased in 26 (32.5%) patients.

The AL CA cohort consisted of 23 patients (see Supplementary data online, *Table S3*). In contrast to the ATTR cohort, patients were younger (58.0 years) and less often male (69.9%). According to the revised Mayo Clinic staging system, 13.0% patients were in stage 1, 30.4% in stage 2, 39.1% in stage 3, and 13.0% in stage 4.^10^ All patients in this cohort received amyloid-specific treatments either before baseline CMR or during the follow-up period. At baseline, patients were most often treated with glucocorticoids (56.5%), daratumumab (47.8%), and/or bortezomib (43.5%).

Median ECV and native T1 times of AL CA patients were 42.6% and 1090 ms, respectively. AL CA patients had moderately hypertrophied LVs (IVS, 15.0 mm; LV mass, 158 g) and presented with preserved LVEF as well as RVEF. At follow-up, ECV increased significantly (*P* = 0.026; *Figure 2* and Supplementary data online, *Figure S2*). Among AL CA patients, ECV decreased in 9 (39.1%) patients and median Δ of ECV was +3.5% (*Figure 3*). Furthermore, we found increases in LVEDV (*P* = 0.010) and RVEDV (*P* = 0.016), while RVEF decreased (*P* = 0.026).

### Impact of treatment on cardiac magnetic resonance imaging parameters in patients with transthyretin amyloidosis

ATTR CA patients were divided into a treatment-naïve cohort, which consisted of patients receiving amyloid-specific medication (excluding epigallocatechin gallate due to the lack of compelling evidence) neither before baseline CMR nor during the follow-up period [*n* = 21 (26.3%); Supplementary data online, *Table S4*], and a treated group consisting of 59 [(73.7%); Supplementary data online, *Table S5*] patients. The treatment-naïve ATTR CA cohort was recruited during a time neither specific TTR-stabilizer nor TTR gene-silencer was available. Median time interval between baseline CMR and start of amyloid-specific treatment in the ATTR cohort was 3.0 months (IQR: 1.0–9.0).

In treatment-naïve patients, ECV increased from 41.8% to 48.8% (*P* < 0.001), whereas it remained stable in the treated cohort (51.2–51.1%, *P* = 0.052). The same could be observed for native T1 time (1071–1100 ms, *P* = 0.044, vs. 1102–1110 ms, *P* = 0.182).

With respect to functional and morphological parameters, we could detect numerically similar deteriorations in LVEF, LVEDV, and RVEDV in both treatment cohorts.

Furthermore, we assessed differences in median Δ of clinical, laboratory, and CMR parameters between treated and treatment-naïve ATTR cohorts. Δ of ECV was significantly higher in untreated patients (+5.7% vs. +2.3%, *P* = 0.004; Supplementary data online, *Table S6*, and Supplementary data online, *Figure S2*). There was no correlation between Δ of ECV and the time interval between baseline CMR and initiation of amyloid-specific treatment (*r* = 0.039, *P* = 0.767).

### Haematological response to treatment in light chain amyloidosis patients and correlation with extracellular volume

Complete remission (CR) 1 year post-immunotherapy was found in 30.4%, very good partial remission (VGPR) in 43.5%, partial response (PR) in 8.7%, and no response (NR) in 8.7% (see Supplementary data online, *Table S3*). No statistical difference (*P* = 0.848) with respect to haematological response was found between patients with decreased and increased follow-up ECV. In detail, patients with decreased follow-up ECV showed CR in 22.2%, VGPR in 66.7%, PR in 11.1%, and NR in 0.0%, while patients with increased follow-up ECV had CR in 38.5%, VGPR in 30.8%, PR in 7.8%, and NR in 15.4%.

### Impact of T1 mapping parameters on outcome

**Figure 4:**
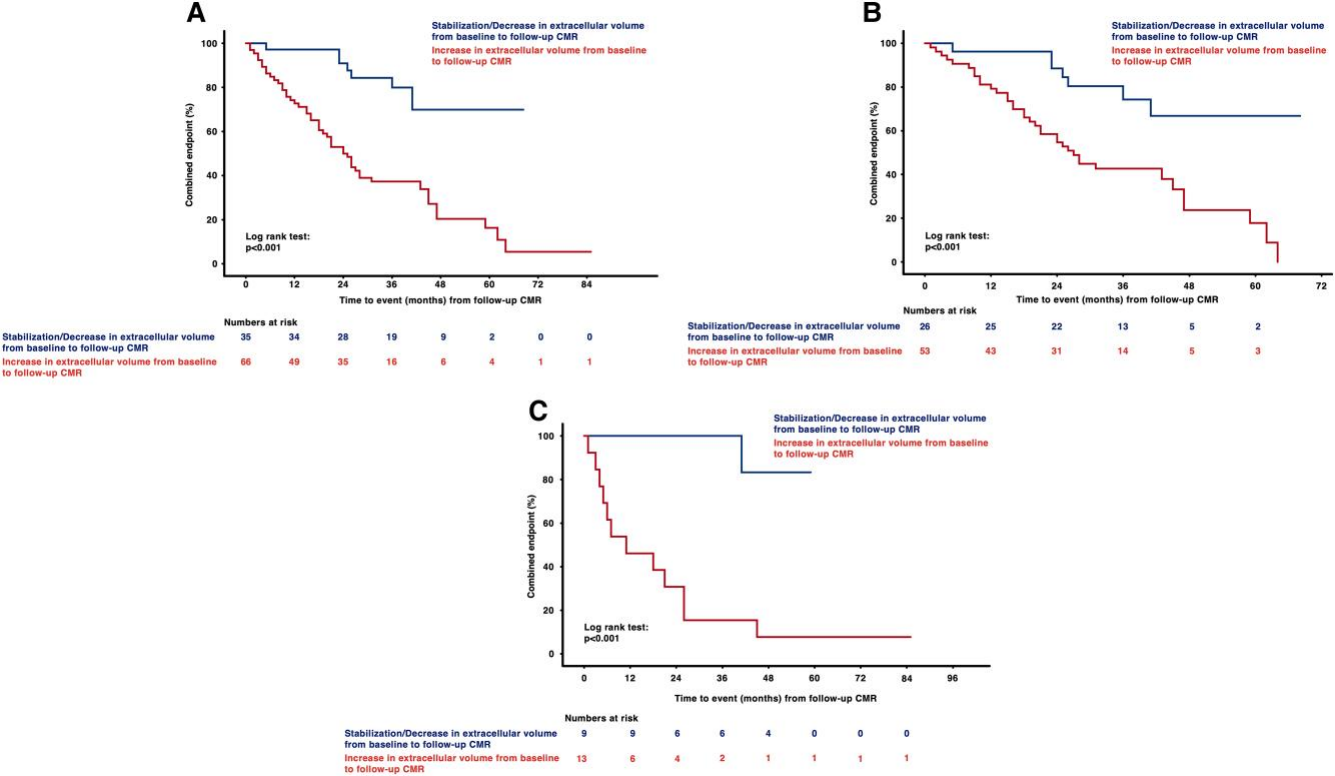
(*A*–*C*) Kaplan–Meier curves for cardiac amyloidosis cohorts stratified according to change in extracellular volume. Patients with increasing extracellular volume (ECV) from baseline to follow-up cardiac magnetic resonance (CMR) imaging had significantly shorter event-free survival (panel *A*, total cohort; panel *B*, transthyretin cohort; and panel *C*, light chain cohort; *P* for all <0.001). Start date for the follow-up period is the date of follow-up CMR.

During a median follow-up period (follow-up CMR to event/database closure) of 27.0 months (18.0–41.0), which did not differ between cohorts (*P* > 0.05 for all), 59 (57.3%) patients reached the combined endpoint of all-cause death or hospitalization for HF. Among ATTR patients, the combined endpoint occurred in 46 (57.5%) patients (all-cause death: *n* = 17, 21.3%; HF hospitalization: *n* = 29, 36.3%), while 13 (56.5%) AL CA patients (all-cause death: *n* = 3, 13.0%; HF hospitalization: *n* = 10, 43.5%) had an outcome events (ATTR CA vs. AL CA: *P* = 0.934). No patient was lost to follow-up. Kaplan–Meier analyses showed that patients in whom ECV increased from baseline to follow-up had significantly shorter event-free survival (*P* < 0.001; *Figure 4A*). This finding was detected irrespective of amyloid subtype (AL: *P* < 0.001, *Figure 4B*; ATTR: *P* < 0.001, *Figure 4C*).

Moreover, in our Cox regression models [Model A: baseline parameters adjusted for baseline NT-proBNP and troponin T as well as time interval between baseline and follow-up CMR, follow-up parameters adjusted for follow-up NT-proBNP and troponin T as well as time between baseline and follow-up CMR; Model B: baseline parameters adjusted for baseline NT-proBNP and troponin T, follow-up parameters adjusted for baseline and follow-up NT-proBNP and troponin T, and Model C: baseline parameters adjusted for baseline Gillmore (ATTR CA cohort) or Mayo Clinic stage (AL CA cohort), follow-up parameters adjusted for follow-up Gillmore (ATTR CA cohort) or Mayo Clinic stage (AL CA cohort); *Table 2*, Supplementary data online, *Tables S8* and *S9*], change of ECV was an independent predictor of outcome in the total CA [Model A: hazard ratio (HR): 1.095, 95% confidence interval (CI): 1.047–1.145, *P* < 0.001; Model B: HR: 1.094, 95% CI: 1.046–1.144, *P* < 0.001], in the ATTR (Model A: HR: 1.073, 95% CI: 1.015–1.134, *P* = 0.013; Model B: HR: 1.074, 95% CI: 1.017–1.134, *P* = 0.010; Model C: 1.064, 95% CI: 1.013–1.117, *P* = 0.013), as well as in the AL CA cohort (Model A: HR: 1.131, 95% CI: 1.041–1.228, *P* = 0.003; Model B: HR: 1.121, 95% CI: 1.033–1.216, *P* = 0.006; Model C: HR: 1.103, 95% CI: 1.018–1.194, *P* = 0.016).

**Table 2:** Cox regression analyses (Model A) for the composite endpoint of all-cause death or heart failure hospitalization in the total, transthyretin, and light chain cardiac amyloidosis cohorts

Variable	Crude hazard ratio	95% Confidence interval	*P* value	Adjusted hazard ratio	95% Confidence interval	*P* value	Adjusted hazard ratio	95% Confidence interval	*P* value
	Univariable regression			Multivariable regression 1			Multivariable regression 2		
**Cardiac magnetic resonance imaging parameters**									
**Total cardiac amyloidosis cohort**									
Baseline native T1 time^a^	1.006	0.999–1.012	0.113	1.008	1.000–1.015	0.0448	n.a		
Follow-up native T1 time^b^	1.004	0.999–1.009	0.155	1.004	0.999–1.010	0.094	1.003	0.998–1.009	0.268
Change of native T1 time^b^	1.003	0.996–1.009	0.396	1.002	0.996–1.009	0.466	1.003	0.996–1.010	0.470
Baseline extracellular volume^a^	1.027	0.998–1.057	0.071	1.027	0.996–1.060	0.093	n.a		
Follow-up extracellular volume^b^	1.024	1.003–1.045	0.026	1.028	1.006–1.051	0.013	1.020	0.996–1.045	0.110
Change of extracellular volume^b^	1.085	1.041–1.130	<0.001	1.113	1.063–1.166	<0.001	1.095	1.047–1.145	<0.001
**Cardiac transthyretin amyloidosis cohort**									
Baseline native T1 time^a^	1.004	0.995–1.013	0.373	1.004	0.994–1.014	0.423	n.a		
Follow-up native T1 time^b^	1.002	0.995–1.009	0.517	1.004	0.997–1.012	0.240	1.002	0.994–1.010	0.673
Change of native T1 time^b^	1.004	0.995–1.013	0.358	1.007	0.997–1.017	0.179	1.006	0.996–1.016	0.267
Baseline extracellular volume^a^	1.030	0.993–1.067	0.114	1.026	0.987–1.065	0.193	n.a		
Follow-up extracellular volume^b^	1.015	0.990–1.040	0.240	1.020	0.994–1.046	0.128	1.004	0.974–1.036	0.775
Change of extracellular volume^b^	1.066	1.015–1.119	0.010	1.094	1.037–1.153	<0.001	1.073	1.015–1.134	0.013
**Cardiac light chain amyloidosis cohort**									
Baseline native T1 time^a^	1.006	0.996–1.016	0.217	1.008	0.995–1.020	0.235	n.a		
Follow-up native T1 time^b^	1.004	0.997–1.012	0.248	1.002	0.992–1.011	0.755	1.004	0.994–1.013	0.437
Change of native T1 time^b^	1.004	0.994–1.014	0.439	0.997	0.985–1.009	0.621	1.000	0.988–1.011	0.959
Baseline extracellular volume^a^	1.019	0.958–1.084	0.557	1.001	0.923–1.085	0.984	n.a		
Follow-up extracellular volume^b^	1.065	1.006–1.126	0.030	1.089	0.987–1.202	0.090	1.133	1.015–1.265	0.026
Change of extracellular volume^b^	1.110	1.033–1.193	0.004	1.114	1.020–1.217	0.017	1.131	1.041–1.228	0.003

Multivariable Model 1: Adjusted for baseline NT-proBNP, troponin T, and time interval between baseline and follow-up cardiac magnetic resonance (CMR) imaging.

Multivariable Model 2: Adjusted for follow-up NT-proBNP, follow-up troponin T, and time interval between baseline and follow-up cardiac magnetic resonance imaging.

^a^Start date for the follow-up period (T0) is the date of baseline CMR.

^b^T0 was date of follow-up CMR.

## Discussion

In our study of 103 CA patients, we could demonstrate that ECV, which predominantly reflects myocardial amyloid load, increases significantly within an 11-month time-period. While this finding was irrespective of amyloid subtype and treatment status, treatment-naïve ATTR patients had the highest increase of median ECV. Changes in ECV were also accompanied by alterations of LV as well as RV dimensional and functional parameters.

Furthermore, our outcome analyses showed that increasing ECV is a predictor of outcome in AL as well as ATTR CA (*Table 2* and Supplementary data online, *Tables S8* and *S9*).

### Extracellular volume in cardiac amyloidosis

In recent years, CMR and T1 mapping have been increasingly used in the diagnostic work-up of CA and quantification of myocardial amyloid load.^2,5,8^ In CA, misfolded proteins infiltrate the extracellular myocardial space, which in turn causes an increase in ECV.^4,11^ In support of this notion, Banypersad et al.^12^ and Martinez-Naharro et al.^13^ could demonstrate markedly elevated ECV values in patients with CA, irrespective of amyloid subtype. However, in comparison to AL, ATTR patients had higher ECVs (40% vs. 56%). These findings are in line with results from our study, where ECV was elevated in both CA cohorts but ATTR patients had higher ECV values.

Despite a growing body of literature on T1 mapping in CA, studies performing serial ECV measurements are scarce.^14^ In our study, median ECV increased significantly during a time span of 12 months, irrespective of amyloid type and treatment status. However, the increase was most pronounced in untreated ATTR patients. Thus, our data suggest that CA is a progressive disease and that current treatments such as TTR stabilizers, small interfering RNA (siRNA), or immuno/chemotherapies are able to slow down but cannot halt the progressive nature of this disease.^15,16^ This is also supported by a recently published study from Fontana et al.^14^ in which ECV increased from 46% to 48% after 12 months of patisiran treatment.

### Change of cardiac dimensions, function, biomarkers, and exercise capacity

In CA, ample amount of data exists regarding alterations of myocardial structure and function, such as bi-ventricular hypertrophy, bi-atrial enlargement, and low stroke volumes despite nominally preserved EF.^17,18,19,20^ However, imaging studies examining longitudinal myocardial functional changes of CA patients are rare, mostly based on echocardiography, and lay focus on the LV.^14,15,21^ In our CMR-based study, LVEF, RVEF, LVEDV, and RVEDV worsened within 12 months of follow-up, changes most likely attributable to the increased deposition of amyloid within the myocardium. These findings are in line with earlier studies by Fontana et al.^22^ and Maurer et al.^23^ in which untreated ATTR patients experienced worsening in LV size and function over time. However, changes in RVEF or RVEDV were not investigated in the above studies. In parallel with a decline in cardiac functional and structural parameters, we could observe increases in NT-proBNP and troponin T in our ATTR CA cohort. However, the deterioration of cardiac biomarkers was less pronounced in the treated-ATTR CA cohort, a finding which is in line with the results of the ATTR-ACT study.^15,22^ Likewise, change in 6-MWD among treated patients was similar to results from the ATTR-ACT trial.^15^

### Extracellular volume as a predictor of outcome in cardiac amyloidosis

Even though the use of T1 mapping and ECV quantification has been recommended for the assessment of CA patients in a joint expert consensus manuscript drafted by the American Society of Nuclear Cardiology, the American College of Cardiology, the American Heart Association, the American Society of Echocardiography, the European Association of Nuclear Medicine, the Heart Failure Society of America, the International Society of Amyloidosis, the Society of Cardiovascular Magnetic Resonance, and the Society of Nuclear Medicine and Molecular Imaging, only a limited number of studies have investigated the prognostic significance of T1 mapping and no studies exist evaluating the predictive power of serial T1 mapping.^13,23,24,25^

Results from our study suggest that change of ECV during the course of the disease might be of greater prognostic relevance than single measurements. However, this finding stands in contrast to previously published work by Banypersad et al. and Martinez-Nahorro et al. In their studies, baseline ECV was associated with adverse outcome in AL as well as ATTR CA patients.^13,23,24^ However, several factors might have contributed to this discrepancy. Firstly, given very similar HRs between Martinez-Nahorros’ and our study [HR: 1.028 (95% CI: 1.008–1.049) vs. HR: 1.026 (95% CI: 0.987–1.065)] the lack of statistical significance might be explained by the smaller ATTR CA patient cohort in our study. Furthermore, this might also be applicable for AL patients, as we included 23 compared with 100 by Banypersad and colleagues. Another explanation could be that in our study all AL patients had definite cardiac involvement, whereas in the study by Banypersad et al. only 53% had definite and further 26% possible cardiac involvement.^23^ Moreover, different endpoints (combined all-cause death/HF hospitalization vs. all-cause death) could also have caused discrepancies in the prognostic relevance of baseline ECV. Additionally, previous studies used smaller regions of interest for ECV quantification.

### Limitations

Major limitations of the present study are heterogeneity of our patient cohort and its relatively small sample size, especially with respect to AL CA patients. Nonetheless, we add a significant number of patients to the existing literature. Due to the single-centre design of our study, a centre-specific bias cannot be excluded. However, limiting data collection to one centre has the advantages of constant quality of work-up, adherence to a constant clinical routine, and constant follow-up. Nonetheless, in a considerable number of patients, 6-MWD was not assessed [missing baseline 6-MWD: 34 (33.0%), missing follow-up 6-MWD: 39 (37.9%)].

However, the present study was focused on changes of CMR parameters, where missing data were <1.0%. Further limitations are the relatively large IQR of the time interval between baseline and follow-up CMR and unknown reasons for missing baseline or follow-up CMR. Another limitation might be a survival bias towards a healthier CA patient population as 27 patients died between baseline and follow-up CMR, and patients without complete follow-up were more often in NYHA class ≥3 and had higher NT-proBNP as well as troponin T levels (see Supplementary data online, *Table S7*). Moreover, T2 times were not assessed in our study. Also, our T1 mapping protocol did not include basal short-axis views, thus reducing our region of interest for ECV quantification. However, basal LV regions were included via T1 maps in four-chamber views. Additionally, the exposure of our cohorts to different therapeutic agents and regimens limits our insight into the natural course of amyloid deposition and ECV expansion in patients with CA. However, this is the first study to systematically perform serial ECV quantification in patients with TTR and AL CA. An additional limitation of our study is that the multivariable Cox regression model for the AL cohort could not be adjusted for treatment response, as only four patients failed to achieve CR or VGPR.

## Conclusions

The present study demonstrates that ECV, quantified by CMR T1 mapping, increases significantly in CA patients over the course of 12 months. While ECV increased, irrespective of amyloid type, our analyses showed the highest increase among untreated ATTR patients. Moreover, the change of ECV was a predictor of adverse outcomes emphasizing the usefulness of serial CMR T1 mapping in the management of CA patients.

## Supplementary data

Supplementary data are available at *European Heart Journal - Cardiovascular Imaging* online.

## Supplementary Material

jead188_Supplementary_Data

## Data Availability

Data will be made available upon reasonable request.
