# Supplementary material for: Myocardial structural and functional changes in cardiac amyloidosis: insights from a prospective observational patient registry
Source: Eur Heart J Cardiovasc Imaging. 2023 Aug 7;25(1):95–104. doi: 10.1093/ehjci/jead188 (PMC10735280; doi:10.1093/ehjci/jead188)
Supplement: jead188_Supplementary_Data [file jead188_supplementary_data.zip › Clean Supplemental figure legends 19.Jul.2023.docx]

**Supplemental figure legends**

**Supplemental figure 1. Regions of interest for extracellular volume quantification.**

Native T1 map of a patient with cardiac transthyretin amyloidosis depicting blood pool and myocardial regions of interest (ROI) in a midventricular short axis slice (A) and a 4-chamber view (B) used for extracellular volume quantification.

**Supplemental figure 2. Median change of extracellular volume in treatment-naïve and treated transthyretin cardiac amyloidosis patients.**

Median increase of extracellular volume between baseline and follow-up was significantly higher in treatment naïve patients [+5.7%, interquartile range (IQR): +3.8% - +7.4%] when compared to treated patients (+2.3%, IQR: -3.1 - +5.1, p=0.004).
