# Supplementary material for: Myocardial structural and functional changes in cardiac amyloidosis: insights from a prospective observational patient registry
Source: Eur Heart J Cardiovasc Imaging. 2023 Aug 7;25(1):95–104. doi: 10.1093/ehjci/jead188 (PMC10735280; doi:10.1093/ehjci/jead188)
Supplement: jead188_Supplementary_Data [file jead188_supplementary_data.zip › Clean_Supplemental methods 19.Jul.2023.docx]

**Supplemental material**

**Diagnosis of cardiac transthyretin amyloidosis**

After publication of the non-invasive diagnostic algorithm by Gillmore et al. in 2016, diagnosis of cardiac ATTR amyloidosis was made if patients had Perugini grade ≥2 myocardial tracer uptake on bone scintigraphy and presence of a paraprotein was ruled out (1). In patients with ambiguous non-invasive test results and before 2016, ATTR amyloidosis was diagnosed if EMB samples stained positive for Congo red, showed apple green birefringence under polarized light and reacted with anti-ATTR antibodies. Gene sequencing was offered to all patients diagnosed with ATTR CA.

**Diagnosis of cardiac light chain amyloidosis**

Cardiac AL amyloidosis was diagnosed if biopsy samples showed Congo red positivity, apple green birefringence under polarized light and reactivity with AL antibodies. In cases of extra-cardiac biopsy samples, cardiac involvement was determined according to current recommendations (2).

**6-minute walk test**

6-minute walk test was performed according to guidelines by the American Thoracic Society indoors on a flat 50m track (3).

**Diagnosis of cardiac light chain amyloidosis**

Cardiac biopsy samples were acquired from the left ventricle (LV) with Bipal® biopsy forceps (Cordis® Corporation, Bridgewater, NJ). Biopsy specimens, irrespective of biopsy site, were fixed in 7.5% buffered formalin for 24h followed by paraffin embedding. Samples were cut in 2µm and 6µm sections using a Leica RM 2255 Microtome (Charleston, SC, USA). 6µm sections were used for Congo red staining, whereas 2µm slices were used for immunohistochemical staining with AmY-kit amyloid antibodies (Martinsried, Germany).

**Cardiac magnetic resonance imaging acquisition and assessment**

CMR indication for baseline as well as follow-up CMR were either due to clinical indication and/or research purposes within the context of our CA patient registry. Neither acquisition protocols, nor the scanner changed throughout the course of the study.

All cardiovascular MRI studies were performed on a 1.5-T system (Avanto FIT; Siemens Medical Solutions, Erlangen, Germany), including late gadolinium enhancement imaging in case of preserved renal function (estimated glomerular filtration rate, >30 mL/min/1.73 m^2^). For cine imaging, steady-state free precision images were used (repetition time msec/echo time msec, 3.2/1.2; flip angle, 64°; voxel size, 1.4 × 1.4 × 6 mm; matrix, 180 × 256 pixels). For late gadolinium chelate enhancement imaging, segmented inversion recovery sequences (700/1.22; flip angle, 50°; voxel size, 1.4 × 1.4 × 8 mm; 146 × 256 matrix) were performed at least 10 minutes after injection of 0.1 mmol/mL gadobutrol (Gadovist; Bayer Vital GmbH, Leverkusen, Germany). We used three long-axis views (one two-chamber, one three-chamber, and one four-chamber view) for calculation of left ventricular global longitudinal strain (GLS). GLS values were averaged from peak values of all 16 American Heart Association segments.

T1 mapping was performed with electrocardiographically triggered MOLLI with a 5(3)3 prototype (5 acquisition heartbeats followed by 3 recovery heartbeats and a further 3 acquisition heartbeats) on a short-axis midcavity slice and with a 4-chamber view. This method generates an inline, pixel-based T1 map by acquiring a series of images over several heartbeats with shifted T1 times, inline motion correction, and inline calculation of the T1 relaxation curve within 1 breath hold. T1-sequence parameters were as follows: starting inversion time (TI) 120 ms, TI increment 80 ms, reconstructed matrix size 256 × 218, measured matrix size 256 × 144 (phase-encoding resolution 66%, phase-encoding field of view 85%). T1 maps were created both before and 15 min after contrast agent application. For post-contrast T1 mapping, a 4(1)3(1)2 prototype was used. To counteract the patchy distribution of amyloid within the myocardium, regions of interest (ROI) in our T1 maps included the whole myocardium (excluding the endocardium) in mid-cavity short axis views and four-chamber views (Supplemental figure 1). T1 times from ROIs were averaged for ECV calculation, which was performed as previously described (4, 5, 6). In all patients, venous blood for conventional hematocrit measurement was drawn when placing the intravenous line for contrast agent administration.

Cardiovascular MRI extracellular volume (ECV) was calculated by using the previously described formula (Δ indicates the change from native and postcontrast mapping results, *T1*_myocardium_ is T1 times of the LV, and *T1*_blood_ is the blood pool)(7):

ECV= (1-hematocrit)*( Δ[1/T1myocardium]/ Δ[1/T1blood])

**Hematological response in light chain amyloidosis**

Complete response (CR) was defined as normal free light chain (FLC) levels with normal kappa/lambda ratio and negative serum as well as urine immunofixation. Reduction of the difference in concentration between the aberrant versus uninvolved class of FLC (dFLC) to <40 mg/L was considered as a very good partial response (VGPR). Partial response (PR) was a >50% reduction in dFLC and no response (NR) less than PR (8).

**Statistical analysis**

Given the fact that currently no consensus exists which change in ECV is clinically meaningful we have computed Kaplan Meier curves dichotomizing our cohort in patients with increasing (≥ +0.1%) or decreasing (≥ -0.1%) ECV. Cox regression models (Model A: baseline parameters adjusted for baseline NT-proBNP and troponin t as well as time interval between baseline and follow-up CMR; follow-up parameters adjusted for follow-up NT-proBNP and troponin T as well as time between baseline and follow-up CMR; Model B: baseline parameters adjusted for baseline NT-proBNP and troponin t; follow-up parameters adjusted for follow-up NT-proBNP and troponin t, Model C: baseline parameters adjusted for baseline Gillmore (ATTR CA cohort), or Mayo Clinic stage (AL CA cohort), follow-up parameters adjusted for follow-up Gillmore (ATTR CA cohort), or Mayo Clinic stage (AL CA cohort) were used to assess the effect of CMR parameters on event-free survival (9, 10):

In order to avoid an immortal time bias in our outcome analyses date of the follow-up CMR represents the start of the follow-up period for Kaplan-Meier curves and Cox regression models when assessing the predictive power of FU parameters and change (Δ) in parameters. Thus, events occurring before follow-up CMR are not included in these models.

**References**

1. Gillmore JD, Maurer MS, Falk RH, Merlini G, Damy T, Dispenzieri A, et al. Nonbiopsy Diagnosis of Cardiac Transthyretin Amyloidosis. Circulation. 2016;133(24):2404-12.

2. Gertz MA, Comenzo R, Falk RH, Fermand JP, Hazenberg BP, Hawkins PN, et al. Definition of organ involvement and treatment response in immunoglobulin light chain amyloidosis (AL): a consensus opinion from the 10th International Symposium on Amyloid and Amyloidosis, Tours, France, 18-22 April 2004. Am J Hematol. 2005;79(4):319-28.

3. ATS statement: guidelines for the six-minute walk test. Am J Respir Crit Care Med. 2002;166(1):111-7.

4. Kammerlander AA, Marzluf BA, Zotter-Tufaro C, Aschauer S, Duca F, Bachmann A, et al. T1 Mapping by CMR Imaging: From Histological Validation to Clinical Implication. JACC: Cardiovascular Imaging. 2016;9(1):14-23.

5. Duca F, Kammerlander AA, Zotter-Tufaro C, Aschauer S, Schwaiger ML, Marzluf BA, et al. Interstitial Fibrosis, Functional Status, and Outcomes in Heart Failure With Preserved Ejection Fraction: Insights From a Prospective Cardiac Magnetic Resonance Imaging Study. Circ Cardiovasc Imaging. 2016;9(12).

6. Kellman P, Wilson JR, Xue H, Ugander M, Arai AE. Extracellular volume fraction mapping in the myocardium, part 1: evaluation of an automated method. J Cardiovasc Magn Reson. 2012;14:63.

7. Kellman P, Wilson JR, Xue H, Ugander M, Arai AE. Extracellular volume fraction mapping in the myocardium, part 1: evaluation of an automated method. Journal of Cardiovascular Magnetic Resonance. 2012;14(1):63.

8. Martinez-Naharro A, Patel R, Kotecha T, Karia N, Ioannou A, Petrie A, et al. Cardiovascular magnetic resonance in light-chain amyloidosis to guide treatment. European Heart Journal. 2022;43(45):4722-35.

9. Kumar S, Dispenzieri A, Lacy MQ, Hayman SR, Buadi FK, Colby C, et al. Revised prognostic staging system for light chain amyloidosis incorporating cardiac biomarkers and serum free light chain measurements. J Clin Oncol. 2012;30(9):989-95.

10. Gillmore JD, Damy T, Fontana M, Hutchinson M, Lachmann HJ, Martinez-Naharro A, et al. A new staging system for cardiac transthyretin amyloidosis. European Heart Journal. 2017;39(30):2799-806.
