# Supplementary material for: Myocardial structural and functional changes in cardiac amyloidosis: insights from a prospective observational patient registry
Source: Eur Heart J Cardiovasc Imaging. 2023 Aug 7;25(1):95–104. doi: 10.1093/ehjci/jead188 (PMC10735280; doi:10.1093/ehjci/jead188)
Supplement: jead188_Supplementary_Data [file jead188_supplementary_data.zip › Clean_Supplemental Tables_03.05.2023.docx]

**Supplemental table 1. Baseline and follow-up characteristics of the complete cardiac amyloidosis cohort.**

| **Variable** | **Baseline**  **(n=103)** | **Follow-up**  **(n=103)** | ***P* value** |
| --- | --- | --- | --- |
| **Comorbidities** | |  |  |
| Non-significant coronary artery disease, n (%) | 10 (9.7) | 11 (10.7) | 1.000 |
| Coronary artery bypass, n (%) | 5 (4.9) | 5 (4.9) | 1.000 |
| Elective coronary stenting, n (%) | 11 (10.7) | 11 (10.7) | 1.000 |
| Myocardial infarction, n (%) | 8 (7.8) | 9 (8.7) | 1.000 |
| Atrial fibrillation/flutter, n (%) | 46 (44.7) | 54 (52.4) | 0.013 |
| Arterial hypertension, n (%) | 60 (58.3) | 38 (36.9) | <0.001 |
| Diabetes mellitus, n (%) | 13 (12.6) | 12 (11.7) | 1.000 |
| Hyperlipidemia, n (%) | 24 (23.3) | 21 (20.4) | 0.453 |
| Polyneuropathy, n (%) | 18 (17.5) | 21 (20.4) | 0.289 |
| **Cardiac medication** | |  |  |
| Angiotensin receptor blocker, n (%) | 33 (32.0) | 20 (19.4) | 0.004 |
| ACE inhibitor, n (%) | 22 (21.4) | 15 (14.6) | 0.180 |
| Beta blocker, n (%) | 48 (46.6) | 36 (35.0) | 0.041 |
| Oral anticoagulant, n (%) | 46 (44.7) | 57 (55.3) | 0.002 |
| Platelet aggregation inhibitor, n (%) | 26 (25.2) | 18 (17.5) | 0.115 |
| Statin, n (%) | 38 (36.9) | 34 (33.0) | 0.344 |
| Loop diuretic, n (%) | 50 (48.5) | 65 (63.1) | 0.003 |
| Thiazide, n (%) | 16 (15.5) | 10 (9.7) | 0.146 |
| Mineralocorticoid receptor antagonist, n (%) | 34 (33.0) | 49 (47.6) | 0.008 |

ACE indicates angiotensin converting enzyme; n.a, not applicable.

**Supplemental table 2. Baseline and follow-up characteristics of the transthyretin amyloidosis cohort.**

| **Variable** | **Baseline**  **(n=80)** | **Follow-up**  **(n=80)** | | ***P* value** | |
| --- | --- | --- | --- | --- | --- |
| **Clinical parameters** |  |  | |  | |
| Age, years (IQR) | 76.0 (71.3 – 80.0) | 78.0 (73.0 – 81.8) | | <0.001 | |
| Sex, male gender, n (%) | 66 (82.5) | 66 (82.5) | | 1.000 | |
| NYHA functional class ≥ III, n (%) | 30 (37.5) | 21 (26.3) | | 0.093 | |
| 6-minute walk test, m (IQR) | 400 (298 – 492) | 400 (322 – 475) | | 0.858 | |
| N-terminal pro brain natriuretic peptide, pg/mL (IQR) | 1835  (827 – 3448) | 2263  (1092 – 3539) | | 0.141 | |
| Troponin t, ng/mL (IQR) | 0.044 (0.029 – 0.065) | 0.053 (0.033 – 0.072) | | 0.043 | |
| eGFR, mL/min/1.73m2 (IQR) | 56.0 (45.3 – 76.3) | 54.9 (44.7 – 67.6) | | 0.079 | |
| Gillmore stage I | 49 (61.3) | 43 (53.8) | | 0.424 | |
| Gillmore stage II | 18 (22.5) | 28 (35.0) | | 0.124 | |
| Gillmore stage III | 12 (15.0) | 8 (10.0) | | 0.386 | |
| **Amyloidosis-specific therapies** |  |  | |  | |
| Tafamidis 20mg, n (%) | 3 (3.8) | 15 (18.8) | | 0.033 | |
| Tafamidis 61mg, n (%) | 1 (1.3) | 41 (51.3) | | <0.001 | |
| Patisiran, n (%) | 0 (0.0) | 2 (2.5) | | 0.500 | |
| Inotersen, n (%) | 0 (0.0) | 1 (1.3) | | 1.000 | |
| **Cardiac magnetic resonance imaging parameters** | |  | |  | |
| Myocardial native T1 time, ms (IQR) | 1100 (1064 – 1141) | 1105 (1077 – 1143) | | 0.034 | |
| Individual change of myocardial T1 time, % (IQR) |  | +9.0 (-17.0 – +29.5) | | n.a | |
| Extracellular volume, % (IQR) | 49.0 (41.0 – 56.8) | 50.5 (44.0 – 61.0) | | <0.001 | |
| Individual change of extracellular volume, % (IQR) |  | +3.5 (-2.0 – +6.7) | | n.a | |
| Interventricular septum, mm (IQR) | 19.0 (16.3 – 22.0) | 19.5 (16.4 – 23.0) | | 0.399 | |
| Left ventricular mass, g (IQR) | 197 (160 – 235) | 201 (155 – 238) | | 0.516 | |
| Left atrial area, cm^2^ (IQR) | 32.0 (27.0 – 38.0) | 32.0 (25.3 – 37.0) | | 0.945 | |
| Right atrial area, cm^2^ (IQR) | 30.5 (25.3 – 37.0) | 30.0 (26.8 – 36.7) | | 0.820 | |
| Left ventricular global longitudinal strain, % (IQR) | -11.9 (-15-0 - -9.1) | -12.3 (-14.5 - -9.0) | 0.487 | |  |
| Left ventricular ejection fraction, % (IQR) | 55.0 (44.3 – 61.0) | 49.1 (44.0 – 58.0) | | 0.033 | |
| Left ventricular cardiac output, L/min (IQR) | 5.7 (4.8 – 6.4) | 5.8 (4.8 – 6.5) | | 0.869 | |
| Left ventricular stroke volume, mL (IQR) | 83.5 (68.8 – 102) | 87.5 (72.3 – 105) | | 0.721 | |
| Left ventricular end-diastolic diameter, mm (IQR) | 44.5 (41.0 – 48.0) | 46.0 (41.0 – 49.0) | | 0.493 | |
| Left ventricular end-diastolic volume, mL (IQR) | 161 (148 – 193) | 170 (154 – 207) | | <0.001 | |
| Right ventricular ejection fraction, % (IQR) | 48.0 (39.0 – 55.4) | 44.5 (37.0 – 50.3) | | 0.009 | |
| Right ventricular cardiac output, L/min (IQR) | 5.3 (4.4 – 6.4) | 5.2 (4.3 – 6.3) | | 0.533 | |
| Right ventricular stroke volume, mL (IQR) | 80.0 (66.8 – 96.8) | 80.5 (65.4 – 96.0) | | 0.960 | |
| Right ventricular end-diastolic diameter, mm (IQR) | 39.0 (34.3 – 45.0) | 41.0 (36.0 – 45.8) | | 0.023 | |
| Right ventricular end-diastolic volume, mL (IQR) | 177 (143 – 206) | 182 (155 – 218) | | <0.001 | |
| Pulmonary artery, mm (IQR) | 28.0 (25.3 – 31.0) | 28.0 (25.0 – 30.9) | | 0.761 | |
| Pleural effusion, n (%) | 20 (25.0) | 29 (36.3) | | 0.049 | |
| Pericardial effusion, n (%) | 31 (38.8) | 35 (43.8) | | 0.541 | |

NYHA indicates New York Heart Association; eGFR, estimated glomerular filtration rate; EGCG; na, not applicable.

**Supplemental table 3. Baseline and follow-up characteristics of the light chain amyloidosis cohort.**

| **Variable** | **Baseline**  **(n=23)** | **Follow-up**  **(n=23)** | ***P* value** |
| --- | --- | --- | --- |
| **Clinical parameters** |  |  |  |
| Age, years (IQR) | 58.0 (53.0 – 74.0) | 60.0 (56.0 – 76.0) | <0.001 |
| Sex, male gender, n (%) | 16 (69.6) | 16 (69.6) | 1.000 |
| NYHA functional class ≥ III, n (%) | 6 (26.1) | 4 (17.4) | 0.625 |
| N-terminal pro brain natriuretic peptide, pg/mL (IQR) | 2062  (421 – 4300) | 1840  (643 – 3701) | 0.357 |
| Troponin t, ng/mL (IQR) | 0.040 (0.022 – 0.069) | 0.040 (0.017 – 0.075) | 0.781 |
| eGFR, mL/min/1.73m2 (IQR) | 57.0 (34.4 – 69.9) | 46.5 (32.0 – 72.0) | 0.107 |
| Revised Mayo Clinic stage I | 3 (13.0) | 5 (21.7) | 0.625 |
| Revised Mayo Clinic stage II | 7 (30.4) | 6 (26.1) | 1.000 |
| Revised Mayo Clinic stage III | 9 (39.1) | 8 (34.8) | 1.000 |
| Revised Mayo Clinic stage IV | 3 (13.0) | 2 (8.7) | 1.000 |
| **Amyloidosis related therapies** |  |  |  |
| Epigallocatechin gallate, n (%) | 1 (4.3) | 1 (4.3) | 1.000 |
| Glucocorticoide, n (%) | 13 (56.5) | 9 (39.1) | 0.344 |
| Daratumubab, n (%) | 11 (47.8) | 12 (52.2) | 1.000 |
| Bortezomib, n (%) | 10 (43.5) | 0 (0.0) | 0.002 |
| Carfilzomib, n (%) | 1 (4.3) | 2 (8.7) | 1.000 |
| Ixazomib, n (%) | 0 (0.0) | 3 (13.0) | 0.250 |
| Cyclophosphamide, n (%) | 3 (13.0) | 0 (0.0) | 0.250 |
| Thalidomide, n (%) | 1 (4.3) | 0 (0.0) | 1.000 |
| Lenalidomide, n (%) | 4 (17.4) | 2 (8.7) | 0.625 |
| Pomalidomide, n (%) | 2 (8.7) | 4 (17.4) | 0.687 |
| Rituximab, n (%) | 0 (0.0) | 0 (0.0) | n.a |
| **Hematological response to treatment at 1 year** | |  |  |
| Complete response, n (%) | n.a | 7 (30.4) | n.a |
| Very good partial response, n (%) | n.a | 10 (43.5) | n.a |
| Partial response, n (%) | n.a | 2 (8.7) | n.a |
| No response, n (%) | n.a | 2 (8.7) | n.a |
| **Cardiac magnetic resonance imaging parameters** | |  |  |
| Myocardial native T1 time, ms (IQR) | 1090 (1043 – 1132) | 1120 (1072 – 1186) | 0.070 |
| Individual change of myocardial T1 time, % (IQR) |  | +26.0 (-21.0 – +62.0) | n.a |
| Extracellular volume, % (IQR) | 42.6 (34.4 – 51.0) | 50.6 (35.7 – 57.4) | 0.026 |
| Individual change of extracellular volume, % (IQR) |  | +3.5 (-1.6 – +9.1) | n.a |
| Interventricular septum, mm (IQR) | 15.0 (14.0 – 17.0) | 15.0 (13.0 – 18.0) | 0.277 |
| Left ventricular mass, g (IQR) | 158 (133 – 210) | 179 (129 – 207) | 0.843 |
| Left atrial area, cm^2^ (IQR) | 29.0 (23.0 – 35.0) | 32.0 (28.0 – 35.0) | 0.385 |
| Right atrial area, cm^2^ (IQR) | 29.0 (24.0 – 31.0) | 30.0 (26.0 – 33.0) | 0.020 |
| Left ventricular global longitudinal strain, % (IQR) | -14.0 (-15.4 - -11.4) | -14.6 (-15.8 - -10.9) | 0.447 |
| Left ventricular ejection fraction, % (IQR) | 62.0 (55.0 – 64.0) | 56.0 (48.8 – 65.0) | 0.201 |
| Left ventricular cardiac output, L/min (IQR) | 5.7 (5.1 – 6.7) | 5.8 (4.9 – 6.4) | 0.831 |
| Left ventricular stroke volume, mL (IQR) | 74.0 (60.0 – 98.0) | 92.0 (67.8 – 108) | 0.031 |
| Left ventricular end-diastolic diameter, mm (IQR) | 46.0 (42.0 – 50.0) | 45.0 (38.0 – 50.0) | 0.480 |
| Left ventricular end-diastolic volume, mL (IQR) | 144 (117 – 184) | 153 (136 – 175) | 0.010 |
| Right ventricular ejection fraction, % (IQR) | 57.0 (46.0 – 62.0) | 50.0 (43.6 – 59.0) | 0.026 |
| Right ventricular cardiac output, L/min (IQR) | 5.3 (5.0 – 6.4) | 5.8 (4.8 – 6.1) | 0.726 |
| Right ventricular stroke volume, mL (IQR) | 82.0 (60.0 – 90.0) | 83.3 (77.0 – 104) | 0.045 |
| Right ventricular end-diastolic diameter, mm (IQR) | 40.0 (36.0 – 43.0) | 41.0 (35.0 – 43.0) | 0.047 |
| Right ventricular end-diastolic volume, mL (IQR) | 154 (135 – 175) | 176 (148 – 183) | 0.016 |
| Pulmonary artery, mm (IQR) | 28.0 (24.0 – 31.0) | 28.0 (25.0 – 33.0) | 0.337 |
| Pleural effusion, n (%) | 8 (34.8) | 6 (26.1) | 0.625 |
| Pericardial effusion, n (%) | 12 (52.2) | 9 (39.1) | 0.508 |

NYHA indicates New York Heart Association; eGFR, estimated glomerular filtration rate; NA, not applicable.

**Supplemental table 4. Baseline and follow-up characteristics of the treatment-naïve* cardiac transthyretin amyloidosis cohort.**

| **Variable** | **Baseline**  **(n=21)** | **Follow-up**  **(n=21)** | | ***P* value** | |
| --- | --- | --- | --- | --- | --- |
| **Clinical parameters** |  |  | |  | |
| Age, years (IQR) | 78.0 (72.0 – 80.0) | 80.0 (74.0 – 83.0) | | 0.052 | |
| NYHA functional class ≥ III, n (%) | 9 (42.9) | 8 (38.1) | | 1.000 | |
| 6-minute walk test, m (IQR) | 330 (190 – 410) | 351 (215 – 435) | | 0.844 | |
| N-terminal pro brain natriuretic peptide, pg/mL (IQR) | 2265  (1095 – 3547) | 2252  (1184 – 4587) | | 0.122 | |
| Troponin t, ng/mL (IQR) | 0.043 (0.0345 – 0.0575) | 0.053 (0.039 – 0.0655) | | 0.330 | |
| eGFR, mL/min/1.73m2 (IQR) | 54.6 (46.1 – 67.4) | 41.8 (37.4 – 54.3) | | 0.848 | |
| Gillmore ATTR stage I, n (%) | 11 (52.4) | 11 (52.4) | | 1.000 | |
| Gillmore ATTR stage II, n (%) | 5 (23.8) | 7 (33.3) | | 0.754 | |
| Gillmore ATTR stage III, n (%) | 5 (23.8) | 3 (14.3) | | 0.687 | |
| **Cardiac magnetic resonance imaging parameters** | |  | |  | |
| Myocardial native T1 time, ms (IQR) | 1071 (1050 – 1109) | 1100 (1070 – 1117) | | 0.044 | |
| Individual change of myocardial T1 time, % (IQR) |  | +11.5 (-3.7 - +37.0) | | n.s | |
| Extracellular volume, % (IQR) | 41.8 (37.4 – 54.3) | 48.8 (40.8 – 62.0) | | <0.001 | |
| Individual change of extracellular volume, % (IQR) |  | +5.7 (+3.8 – +7.4) | | n.a | |
| Interventricular septum, mm (IQR) | 19.0 (18.0 – 22.0) | 19.0 (16.5 – 23.0) | | 0.984 | |
| Left ventricular mass, g (IQR) | 194 (160 – 229) | 211 (155 – 231) | | 0.296 | |
| Left atrial area, cm^2^ (IQR) | 28.0 (24.5 – 34.5) | 29.3 (27.0 – 34.0) | | <0.001 | |
| Right atrial area, cm^2^ (IQR) | 28.0 (22.0 – 36.5) | 29.0 (22.8 – 37.0) | | <0.001 | |
| Left ventricular global longitudinal strain, % (IQR) | -12.8 (-15.3 – -8.6) | | -11.4 (-14.3 – -8.3) | 0.170 |  |
| Left ventricular ejection fraction, % (IQR) | 55.0 (41.5 – 60.5) | 48.0 (44.9 – 56.5) | | 0.627 | |
| Left ventricular cardiac output, L/min (IQR) | 6.0 (4.4 – 6.8) | 5.8 (5.1 – 6.4) | | 0.444 | |
| Left ventricular stroke volume, mL (IQR) | 81.0 (56.5 – 96.5) | 82.0 (75.0 – 97.5) | | 0.281 | |
| Left ventricular end-diastolic diameter, mm (IQR) | 44.0 (40.5 – 48.5) | 46.0 (40.5 – 49.5) | | 0.983 | |
| Left ventricular end-diastolic volume, mL (IQR) | 160 (140 – 177) | 167 (155 – 191) | | 0.008 | |
| Right ventricular ejection fraction, % (IQR) | 41.0 (37.5 – 57.8) | 43.0 (39.5 – 49.0) | | 0.695 | |
| Right ventricular cardiac output, L/min (IQR) | 5.2 (4.0 – 6.7) | 5.3 (4.7 – 6.4) | | 0.695 | |
| Right ventricular stroke volume, mL (IQR) | 80.0 (51.0 – 88.5) | 83.0 (65.6 – 89.5) | | 0.509 | |
| Right ventricular end-diastolic diameter, mm (IQR) | 38.0 (35.5 – 41.5) | 39.0 (36.0 – 43.2) | | 0.170 | |
| Right ventricular end-diastolic volume, mL (IQR) | 165 (143 – 201) | 177 (163 – 201) | | 0.014 | |
| Pulmonary artery, mm (IQR) | 28.0 (25.5 – 31.0) | 27.0 (26.0 – 30.5) | | 0.913 | |
| Pleural effusion, n (%) | 8 (38.1) | 9 (42.9) | | 1.000 | |
| Pericardial effusion, n (%) | 12 (57.1) | 13 (61.9) | | 1.000 | |

NYHA indicates New York Heart Association; eGFR, estimated glomerular filtration rate; not applicable.
* including 7 patients receiving epigallocatechin-3-gallate

**Supplemental Table 5. Baseline and follow-up characteristics of the treated cardiac transthyretin amyloidosis cohort.**

| **Variable** | **Baseline**  **(n=59)** | | **Follow-up**  **(n=59)** | ***P* value** | |
| --- | --- | --- | --- | --- | --- |
| **Clinical parameters** |  | |  |  | |
| Age, years (IQR) | 76.0 (70.0 – 80.0 | | 78.0 (72.0 – 81.0) | 0.446 | |
| NYHA functional class ≥ III, n (%) | 21 (36.2) | 13 (22.0) | | 0.077 | |
| 6-minute walk test, m (IQR) | 433 (330 – 500) | | 410 (348 – 503) | 0.981 | |
| N-terminal pro brain natriuretic peptide, pg/mL (IQR) | 1792  (787 – 3459) | | 2273  (1077 – 3205) | 0.464 | |
| Troponin t, ng/mL (IQR) | 0.045 (0.029 – 0.066) | | 0.052 (0.030 – 0.073) | 0.056 | |
| eGFR, mL/min/1.73m2 (IQR) | 56.6 (46.4 – 80.5) | | 54.4 (44.2 – 67.7) | 0.029 | |
| Gillmore ATTR stage I, n (%) | 38 (65.5) | | 33 (55.9) | 0.332 | |
| Gillmore ATTR stage II, n (%) | 13 (22.4) | | 21 (35.6) | 0.143 | |
| Gillmore ATTR stage III, n (%) | 7 (12.1) | | 5 (8.5) | 0.687 | |
| **Amyloidosis related therapies** |  | |  |  | |
| Tafamidis, n (%) | 3 (5.1) | | 55 (93.2) | <0.001 | |
| Patisiran, n (%) | 0 (0.0) | | 2 (3.4) | n.a | |
| Inotersen, n (%) | 0 (0.0) | | 1 (1.7) | n.a | |
| Epigallocatechin gallate, n (%) | 23 (39.0) | | 43 (72.9) | <0.001 | |
| **Cardiac magnetic resonance imaging parameters** | | |  |  | |
| Myocardial native T1 time, ms (IQR) | 1102 (1073 – 1143) | | 1110 (1080 – 1145) | 0.183 | |
| Individual change of myocardial T1 time, % (IQR) |  | | +9 (-19.0 - +28.0) | n.a | |
| Extracellular volume, % (IQR) | 51.2 (41.6 – 57.5) | | 51.1 (44.1 – 60.0) | 0.052 | |
| Individual change of extracellular volume, % (IQR) |  | | +2.3 (-3.1 – +5.1) | n.a | |
| Interventricular septum, mm (IQR) | 19.0 (16.0 – 22.0) | | 19.9 (16.2 – 23.0) | 0.297 | |
| Left ventricular mass, g (IQR) | 197 (159 – 239) | | 195 (155 – 239) | 0.844 | |
| Left atrial area, cm^2^ (IQR) | 32.0 (28.0 – 39.0) | | 33.0 (29.0 – 38.0) | <0.001 | |
| Right atrial area, cm^2^ (IQR) | 31.0 (26.0 – 37.0) | | 31.0 (27.9 – 36.0) | <0.001 | |
| Left ventricular global longitudinal strain, % (IQR) | -11.9 (-14.5 – -9.2) | | -12.8 (-14.6 – -9.5) | 0.985 |  |
| Left ventricular ejection fraction, % (IQR) | 55.0 (45.0 – 61.0) | | 49.1 (43.0 – 58.0) | 0.032 | |
| Left ventricular cardiac output, L/min (IQR) | 5.6 (4.8 – 6.3) | | 5.7 (4.5 – 6.5) | 0.426 | |
| Left ventricular stroke volume, mL (IQR) | 87.0 (76.0 – 102) | | 88.8 (71.0 – 106) | 0.789 | |
| Left ventricular end-diastolic diameter, mm (IQR) | 45.0 (41.0 – 48.0) | | 46.0 (41.0 -49.0) | 0.330 | |
| Left ventricular end-diastolic volume, mL (IQR) | 162 (150 – 207) | | 170 (151 – 210) | 0.020 | |
| Right ventricular ejection fraction, % (IQR) | 49.0 (40.0 – 55.0) | | 45.6 (36.0 – 51.0) | 0.005 | |
| Right ventricular cardiac output, L/min (IQR) | 5.3 (4.4 – 6.2) | | 5.1 (4.1 – 6.3) | 0.282 | |
| Right ventricular stroke volume, mL (IQR) | 79.0 (70.0 – 99.0) | | 79.0 (64.0 – 99.0) | 0.524 | |
| Right ventricular end-diastolic diameter, mm (IQR) | 40.0 (34.0 – 46.0) | | 42.0 (36.0 – 46.0) | 0.077 | |
| Right ventricular end-diastolic volume, mL (IQR) | 177 (143 – 216) | | 182 (153 – 232) | 0.016 | |
| Pulmonary artery, mm (IQR) | 28.0 (25.0 – 31.0) | | 28.0 (25.0 – 31.0) | 0.797 | |
| Pleural effusion, n (%) | 12 (20.3) | | 20 (33.9) | 0.039 | |
| Pericardial effusion, n (%) | 19 (32.2) | | 22 (37.3) | 0.648 | |

NYHA indicates New York Heart Association; eGFR, estimated glomerular filtration rate; n.a, not applicable.

**Supplemental Table 6. Differences in clinical and cardiac magnetic resonance imaging parameters between treatment-naïve* and treated cardiac transthyretin amyloidosis patients.**

| **Variable** | **Treatment- naïve**  **ATTR amyloidosis cohort** | **Treated ATTR amyloidosis cohort** | ***P* value** |
| --- | --- | --- | --- |
| **Clinical parameters** |  |  |  |
| Δ6-minute walk test, m (IQR) | 6.0 (-29.0 – 81.5) | 0.0 (-43.5 – 46.5) | 0.557 |
| ΔN-terminal pro brain natriuretic peptide, pg/mL (IQR) | 294 (-156 – 1910) | 84.2 (-549 – 1172) | 0.246 |
| ΔTroponin t, ng/mL (IQR) | 0.030 (-0.002 – 0.011) | 0.001 (-0.002 – 0.207) | 0.746 |
| ΔeGFR, mL/min/1.73m2 (IQR) | -2.3 (-7.4 – 13.7) | -2.1 (-13.0 – 3.3) | 0.277 |
| **Cardiac magnetic resonance imaging parameters** |  |  |  |
| ΔMyocardial native T1 time, ms (IQR) | +11.5 (-3.8 – +37.0) | +9.0 (-19.0 – +28.0 | 0.385 |
| ΔExtracellular volume, % (IQR) | +5.7 (+3.8 - +7.4) | +2.3 (-3.1 – +5.1) | 0.004 |
| ΔInterventricular septum, mm (IQR) | 1.0 (-1.5 – 1.5) | 1.0 (-1.1 – 3.0) | 0.531 |
| ΔLeft ventricular mass, g (IQR) | 7.0 (-11.0 – 26.5) | 0.0 (-18.4 – 19.0) | 0.431 |
| ΔLeft atrial area, cm^2^ (IQR) | 1.0 (-4.5 – 3.5) | 0.0 (-2.6 – 3.0) | 0.738 |
| ΔRight atrial area, cm^2^ (IQR) | 1.1 (-2.5 – 3.5) | 0.0 (-3.7 – 3.0) | 0.669 |
| ΔLeft ventricular global longitudinal strain, % (IQR) | 1.1 (-1.2 – 2.8) | -0.5 (-1.5 – 2.3) | 0.251 |
| ΔLeft ventricular ejection fraction, % (IQR) | -2.0 (-11.2 – 9.9) | -2.3 (-9.4 – 2.0) | 0.726 |
| ΔLeft ventricular cardiac output, L/min (IQR) | -0.2 (-1.3 – 0.1) | 0.01 (-0.7 – 0.9) | 0.258 |
| ΔLeft ventricular stroke volume, mL (IQR) | -5.0 (-20.5 – 12.0) | 0.0 (-13.0 – 13.0) | 0.299 |
| ΔLeft ventricular end-diastolic diameter, mm (IQR) | -0.9 (-4.4 – 5.0) | 0.2 (-2.0 – 3.0) | 0.480 |
| ΔLeft ventricular end-diastolic volume, mL (IQR) | 15.0 (0.5 – 38.8) | 5.0 (-4.0 – 24.0) | 0.229 |
| ΔRight ventricular ejection fraction, % (IQR) | -1.0 (-8.3 – 8.5) | -3.5 (-9.7 – 2.0) | 0.336 |
| ΔRight ventricular cardiac output, L/min (IQR) | 0.3 (-1.2 – 1.1) | -0.1 (-1.1 – 0.8) | 0.293 |
| ΔRight ventricular stroke volume, mL (IQR) | 7.0 (-17.5 – 20.6) | 0.0 (-15.0 – 9.5) | 0.358 |
| ΔRight ventricular end-diastolic diameter, mm (IQR) | 1.4 (-1.0 – 6.5) | 2.0 (-3.0 – 6.0) | 0.908 |
| ΔRight ventricular end-diastolic volume, mL (IQR) | 11.0 (-1.8 – 43.0) | 9.0 (-14.0 – 36.9) | 0.434 |
| ΔPulmonary artery, mm (IQR) | 0.0 (-2.5 – 2.9) | 0.0 (-2.5 – 2.0) | 0.886 |

Δ indicates change from baseline to follow-up; eGFR, estimated glomerular filtration rate.
* including 7 patients receiving epigallocatechin-3-gallate.

**Supplemental table 7. Characteristics of the final study cohort and patients who did not undergo follow-up cardiac magnetic resonance imaging.**

| **Variable** | **Baseline &  follow-up CMR**  **(n=103)** | **No follow-up CMR**  **(n=88)** | ***P* value** |
| --- | --- | --- | --- |
| **Clinical parameters** |  |  |  |
| Age, years (IQR) | 75.0 (68.0 – 79.0) | 74.5 (67.3 – 81.0) | 0.727 |
| Sex, male gender, n (%) | 82 (79.6) | 57 (64.8) | 0.022 |
| NYHA functional class ≥ III, n (%) | 36 (35.0) | 45 (51.1) | 0.005 |
| 6-minute walk test, m (IQR) | 400 (296 – 480) | 357 (293 – 406) | 0.220 |
| N-terminal pro brain natriuretic peptide, pg/mL (IQR) | 1917  (787 – 3542) | 4076  (1659 - 10224) | <0.001 |
| Troponin t, ng/mL (IQR) | 0.043 (0.029 – 0.065) | 0.064 (0.030 – 0.111) | 0.010 |
| eGFR, mL/min/1.73m2 (IQR) | 56.5 (42.4 – 75.0) | 47.9 (38.2 – 62.9) | 0.005 |
| **Cardiac magnetic resonance imaging parameters** | |  |  |
| Extracellular volume, % (IQR) | 48.0 (40.0 – 55.3) | 46.6 (36.1 – 55.0) | 0.705 |
| Interventricular septum, mm (IQR) | 18.0 (15.0 – 21.0) | 18.0 (14.0 – 20.0) | 0.192 |
| Left ventricular mass, g (IQR) | 194 (154 – 229) | 174 (137 - 211) | 0.063 |
| Left atrial area, cm^2^ (IQR) | 31.0 (26.0 – 37.0) | 31.0 (26.0 – 35.0) | <0.001 |
| Right atrial area, cm^2^ (IQR) | 30.0 (25.0 – 36.0) | 27.0 (22.0 – 33.8) | 0.029 |
| Left ventricular ejection fraction, % (IQR) | 56.0 (47.0 – 62.0) | 55.0 (46.2 – 64.5) | 0.934 |
| Left ventricular cardiac output, L/min (IQR) | 5.7 (4.9 – 6.5) | 5.3 (4.3 – 6.2) | 0.124 |
| Right ventricular ejection fraction, % (IQR) | 49.0 (41.0 – 58.0) | 48.0 (39.0 – 56.0) | 0.404 |
| Right ventricular cardiac output, L/min (IQR) | 5.3 (4.5 – 6.4) | 5.1 (4.3 – 6.4) | 0.802 |
| Pulmonary artery, mm (IQR) | 28.0 (25.0 – 31.0) | 28.0 (25.3 – 32.0) | 0.695 |
| Pleural effusion, n (%) | 28 (27.2) | 40 (45.5) | 0.009 |
| Pericardial effusion, n (%) | 43 (41.7) | 37 (42.0) | 0.967 |

CMR indicates cardiac magnetic resonance imaging; NYHA, New York Heart Association; eGFR, estimated glomerular filtration rate.

**Supplemental table 8. Cox regression analyses (Model B) for the composite endpoint of all-cause death or heart failure hospitalization in the total, transthyretin and light chain cardiac amyloidosis cohorts.**

|  | **Crude hazard ratio** | **95% Confidence interval** | | **P value** | **Adjusted hazard ratio** | **95% Confidence interval** | **P value** |
| --- | --- | --- | --- | --- | --- | --- | --- |
|  | **Univariable regression** | | | | **Multivariable regression** | | |
| **Total cardiac amyloidosis cohort** | | |  | |  |  |  |
| Baseline native T1 time * | 1.006 | 0.999 – 1.012 | 0.113 | | 1.007 | 1.000 – 1.015 | 0.048 |
| Follow-up native T1 time † | 1.004 | 0.999 – 1.009 | 0.155 | | 1.003 | 0.998 – 1.009 | 0.278 |
| Change of native T1 time † | 1.003 | 0.996 – 1.009 | 0.396 | | 1.003 | 0.996 – 1.010 | 0.474 |
| Baseline extracellular volume * | 1.027 | 0.998 – 1.057 | 0.071 | | 1.028 | 0.997 – 1.059 | 0.081 |
| Follow-up extracellular volume † | 1.024 | 1.003 – 1.045 | 0.026 | | 1.020 | 0.995 – 1.044 | 0.113 |
| Change of extracellular volume † | 1.085 | 1.041 – 1.130 | <0.001 | | 1.094 | 1.046 – 1.144 | <0.001 |
| **Cardiac transthyretin amyloidosis cohort** | | |  | |  |  |  |
| Baseline native T1 time * | 1.004 | 0.995 – 1.013 | 0.373 | | 1.005 | 0.996 – 1.015 | 0.292 |
| Follow-up native T1 time † | 1.002 | 0.995 – 1.009 | 0.517 | | 1.001 | 0.993 – 1.009 | 0.767 |
| Change of native T1 time † | 1.004 | 0.995 – 1.013 | 0.358 | | 1.006 | 0.996 – 1.016 | 0.233 |
| Baseline extracellular volume * | 1.030 | 0.993 – 1.067 | 0.114 | | 1.030 | 0.993 – 1.067 | 0.114 |
| Follow-up extracellular volume † | 1.015 | 0.990 – 1.040 | 0.240 | | 1.004 | 0.975 – 1.034 | 0.771 |
| Change of extracellular volume † | 1.066 | 1.015 – 1.119 | 0.010 | | 1.074 | 1.017 – 1.134 | 0.010 |
| **Cardiac light chain amyloidosis cohort** | | |  | |  |  |  |
| Baseline native T1 time * | 1.006 | 0.996 – 1.016 | 0.217 | | 1.008 | 0.995 – 1.020 | 0.219 |
| Follow-up native T1 time † | 1.004 | 0.997 – 1.012 | 0.248 | | 1.004 | 0.994 – 1.014 | 0.439 |
| Change of native T1 time † | 1.004 | 0.994 – 1.014 | 0.439 | | 1.002 | 0.990 – 1.014 | 0.770 |
| Baseline extracellular volume * | 1.019 | 0.958 – 1.084 | 0.557 | | 1.004 | 0.929 – 1.086 | 0.918 |
| Follow-up extracellular volume † | 1.065 | 1.006 – 1.126 | 0.030 | | 1.109 | 1.010 – 1.218 | 0.030 |
| Change of extracellular volume † | 1.110 | 1.033 – 1.193 | 0.004 | | 1.121 | 1.033 – 1.216 | 0.006 |

* Adjusted for baseline NT-proBNP and troponin t. Start date for the follow-up period (T0) is the date of baseline cardiac magnetic resonance imaging (CMR) imaging.

† Adjusted for follow-up NT-proBNP and troponin t. T0 for this analysis was date of follow-up CMR.

N-terminal pro brain natriuretic peptide and troponin t were divided into quartiles.

**Supplemental table 9. Cox regression analyses (Model C) for the composite endpoint of all-cause death or heart failure hospitalization in the transthyretin and light chain cardiac amyloidosis cohorts.**

| **Variable** | **Crude hazard ratio** | **95% Confidence interval** | | **P value** | **Adjusted hazard ratio*** | **95% Confidence interval** | **P value** |
| --- | --- | --- | --- | --- | --- | --- | --- |
|  | **Univariable regression** | | | | **Multivariable regression** | | |
| **Cardiac transthyretin amyloidosis cohort** | | |  | |  |  |  |
| Baseline native T1 time * | 1.004 | 0.995 – 1.013 | 0.373 | | 1.003 | 0.994 – 1.013 | 0.467 |
| Follow-up native T1 time † | 1.002 | 0.995 – 1.009 | 0.517 | | 1.002 | 0.995 – 1.009 | 0.529 |
| Change of native T1 time † | 1.004 | 0.995 – 1.013 | 0.358 | | 1.004 | 0.996 – 1.013 | 0.320 |
| Baseline extracellular volume * | 1.030 | 0.993 – 1.067 | 0.114 | | 0.862 | 0.992 – 1.064 | 0.127 |
| Follow-up extracellular volume † | 1.015 | 0.990 – 1.040 | 0.240 | | 1.014 | 0.990 – 1.039 | 0.248 |
| Change of extracellular volume † | 1.066 | 1.015 – 1.119 | 0.010 | | 1.064 | 1.013 – 1.117 | 0.013 |
| **Cardiac light chain amyloidosis cohort** | | |  | |  |  |  |
| Baseline native T1 time ** | 1.006 | 0.996 – 1.016 | 0.217 | | 1.010 | 0.997 – 1.023 | 0.123 |
| Follow-up native T1 time †† | 1.004 | 0.997 – 1.012 | 0.248 | | 1.003 | 0.994 – 1.012 | 0.486 |
| Change of native T1 time †† | 1.004 | 0.994 – 1.014 | 0.439 | | 1.001 | 0.990 – 1.013 | 0.847 |
| Baseline extracellular volume ** | 1.019 | 0.958 – 1.084 | 0.557 | | 1.022 | 0.942 – 1.109 | 0.599 |
| Follow-up extracellular volume †† | 1.065 | 1.006 – 1.126 | 0.030 | | 1.074 | 0.998 – 1.155 | 0.056 |
| Change of extracellular volume †† | 1.110 | 1.033 – 1.193 | 0.004 | | 1.103 | 1.018 – 1.194 | 0.016 |

* Adjusted for baseline Gillmore stage. Start date for the follow-up period (T0) is the date of baseline cardiac magnetic resonance imaging (CMR) imaging.

† Adjusted for follow-up Gillmore stage. T0 is the date of follow-up CMR.

** Adjusted for baseline Mayo Clinic stage. T0 is the date of baseline CMR.

†† Adjusted for follow-up Mayo Clinic stage. T0 is the date of follow-up CMR.
