# Supplementary figures and images for: Myocardial structural and functional changes in cardiac amyloidosis: insights from a prospective observational patient registry
Source: Eur Heart J Cardiovasc Imaging. 2023 Aug 7;25(1):95–104. doi: 10.1093/ehjci/jead188 (PMC10735280; doi:10.1093/ehjci/jead188)

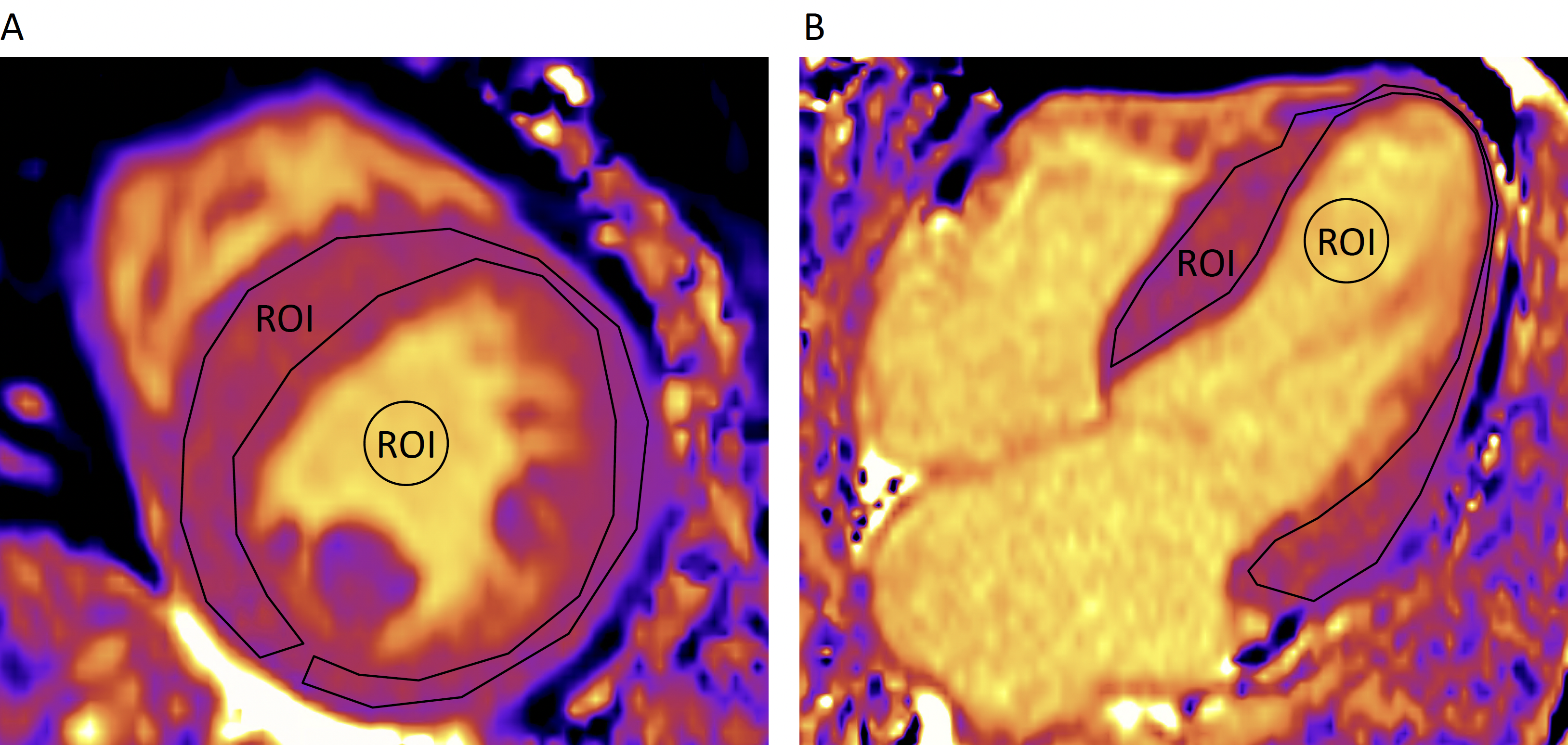

Supplement: jead188_Supplementary_Data [file jead188_supplementary_data.zip › Supplemental figure 1_03.05.2023.tiff]

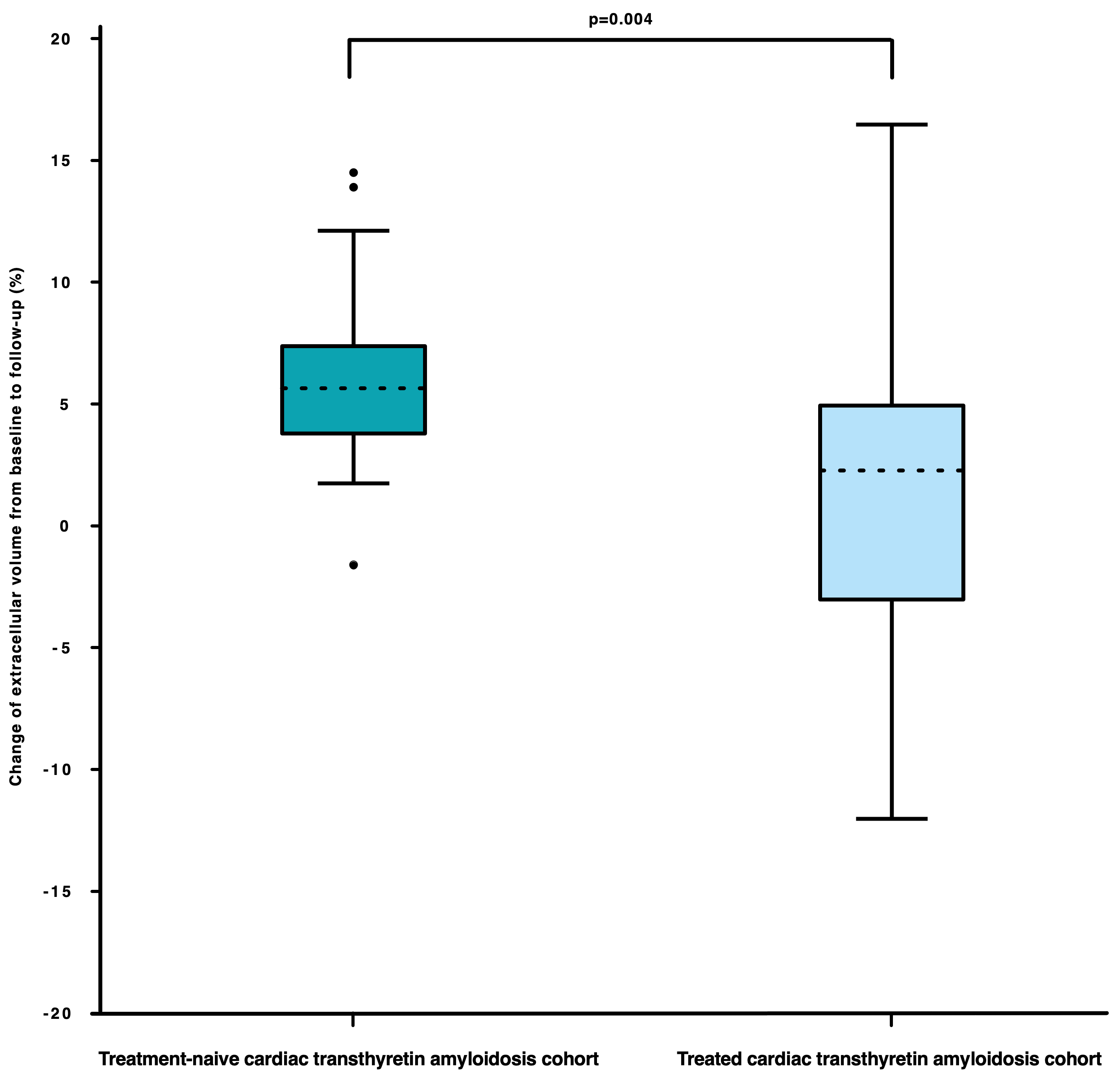

Supplement: jead188_Supplementary_Data [file jead188_supplementary_data.zip › Supplemental figure 2_02.05.2023.tiff]
